# Post-ischemic continuous infusion of erythropoeitin enhances recovery of lost memory function after global cerebral ischemia in the rat

**DOI:** 10.1186/1471-2202-14-27

**Published:** 2013-03-12

**Authors:** Johan Undén, Carin Sjölund, John-Kalle Länsberg, Tadeusz Wieloch, Karsten Ruscher, Bertil Romner

**Affiliations:** 1 Department of Perioperative Medicine and Intensive Care, Skane University Hospital, Malmö, S-20502, Sweden; 2 Department of Clinical Sciences, Division of Neurosurgery, Laboratory for Experimental Brain Research, Wallenberg Neuroscience Center, Lund University, BMC A13, Lund, S-22184, Sweden; 3 Department of Clinical Sciences, Section for Paediatrics, The BUT Team, Lund University, Lund, S-22185, Sweden; 4 Department of Clinical Sciences, Division of Neurosurgery, Lund University, Lund, S-22185, Sweden

## Abstract

**Background:**

Erythropoietin (EPO) and its covalently modified analogs are neuroprotective in various models of brain damage and disease. We investigated the effect on brain damage and memory performance, of a continuous 3-day intravenous infusion of EPO, starting 20 min after a transient 10 minute period of global cerebral ischemia in the rat.

**Results:**

We found no effect on selective neuronal damage in the CA1 region of the hippocampus, neocortical damage and damage to the striatum assessed at 7 days after ischemia. Also, no differences were observed in sensori-motor scores between EPO treated and saline treated ischemic animals. In contrast, memory performance was significantly improved in the EPO treated group. Saline treated injured animals (n = 7) failed in a test assessing recovery of spatial memory (6/6 and 5/6), while EPO treated animals had few and none failures (0/7 and 1/7).

**Conclusion:**

We conclude that although post-ischemic treatment with EPO is not neuroprotective in a model of cardiac arrest brain ischemia, its markedly positive effect on brain plasticity and recovery of memory function warrants consideration as treatment of cardiac arrest patients.

## Background

Erythropoietin (EPO), a cytokine and growth factor and one of the principal modulators of erythropoiesis [1,2], has recently been recognized for its broad applicability in medicine [3]. The EPO gene is regulated by hypoxia inducing factor (HIF), where it stimulates erythropeoisis and proliferation of parenchymal cells, by acting on the EPO receptor (EpoR) coupled to PI3-kinase, JAK/STAT and NF-κB pathways [4-6]. In the brain, EPO is neuroprotective and has also brain restorative properties in different models of acute neuronal death and neurodegenerative diseases, through effects on neurons and astrocytes by multimodal actions [7]. In models of stroke, spinal cord injury, traumatic brain injury, brain edema, EPO is clearly neuroprotective [8-11]. In models of focal ischemia in the rat EPO is both protective, i.e. decreases infarct size but also enhances brain plasticity in rat and mice [12]. Here the carbamylated form of EPO, CEPO, that lacks erythropoietic activity, can be administered up to 7 days after MCAO with a significant improvement of sensori-motor function but without affecting infarct size. This strongly suggests that EPO enhances plasticity when administered in the subacute phase after stroke. Indeed EPO also stimulates cognition and enhances LTP [13].

In models of global ischemia, mimicking cardiac arrest, EPO is protective though only if applied prior to or 20 minutes after ischemia and only when administered through the intracerebroventricular (i.c.v.) route [14]. If provided intravenously within 5 minutes after recovery of spontanuous cardiac activity and then subsequently at 1 and 3 days of recovery after the ischemic insult, no beneficial effects are seen on neither morphological outcome, markers of cell death or gross sensori-motor scores [15]. The aim of the present investigation was to study the effect of EPO treatment as a continuous intravenous administration in a model of global ischemia starting the treatment 20 minutes after reperfusion. We choose a route and time of administration that can be expected in a possible treatment of cardiac arrest patients. We employed the two vessel occlusion (2-VO) model of global cerebral ischemia [16] and assessed the histological outcome and sensori-motor and memory performance after ischemia.

## Methods

### Surgical procedures

The animal experiments were performed on male Wistar rats, weighing 300-350 g (Møllegaard’s Breeding Center, Copenhagen, Denmark), which were fasted overnight with free access to tap water. The experiments were approved by the Malmö/Lund Ethical Committee on Animal Experiments and were in accordance with the guidelines of the National Institute of Health (NIH). All efforts were made to minimize animal suffering and to reduce the number of animals used.

The two-vessel occlusion (2-VO) model of global cerebral ischemia was used [16]. In brief, the animals were initially anaesthetized with 3.5-4% isoflurane in N_2_O/O_2_ (70:30) and then intubated and connected to a small animal respirator. The isoflurane concentration was reduced to 1.5% for the remainder of the surgery. The tail artery and vein were cannulated for blood pressure recordings, blood sampling and drug infusions. The right jugular vein was used to insert a soft silastic catheter into the superior caval vein for rapid withdrawal of blood and reduction of the blood pressure to 45–50 mmHg during ischemia. Both common carotid arteries were dissected and encircled with loose ligatures. In addition, EEG electrodes were placed into the temporal muscles and EEG activity recorded. Thereafter, both common carotid arteries were reversible clamped for 10 minutes. At the end of surgery, isoflurane concentration was reduced to 0.5-1% and the ventilation and O_2_ supply was adjusted to an arterial P_CO2_ of 35–40 mmHg and a P_O2_ of close to 100 mmHg. Heparin (90 IU.kg^-1^) was given prior to the first blood gas measurement. During the experiment, animals were paralyzed with vecuronium bromide (2 mg.h^-1^). The animals’ head and body temperature were kept close to 37°C using a heating lamp and a thermostat-regulated pad.

Immediately after ischemia, 0.5 ml of a 0.6 mol/L NaHCO_3_ solution was given intravenously to neutralize systemic acidosis, and isoflurane supply was discontinued. When the animals regained spontaneous breathing they were extubated and disconnected from the respirator and were then housed in cages with free access to tap water and pellet food. Sham-operated animals were treated in the same way as those in the ischemia group except for the occlusion of the carotid arteries and the reduction of blood pressure. In total 23 rats were operated, thereof 2 rats were excluded immediately after surgery due to surgery problems, one animal died on day 3 after surgery.

### Randomization, EPO administration and EPO analysis

Every other animal was randomized into the treatment groups and received either an intravenous bolus injection of EPO (Neorecormon, Roche, Switzerland) immediately after surgery calculated based on the following equation: 80 IU multiplied with the volume of distribution (VD; 0.057 mL/g BW). Thereafter, maintenance intravenous infusion was performed at 160 IU per hour via an osmotic minipump (mini-osmotic alzet pump2001 D) for 72 h or an intravenous saline injection immediately after surgery and saline infusion via an osmotic minipump for additional 72 h (mini-osmotic alzet pump 1003D). Serum was analyzed for EPO 10 to 15 minutes after the bolus injection and 5 and 72 h after the surgery using an automated ELISA system (Quantikine® IVD® ELISA, R&D Systems, UK).

### Histopathological procedures

For histopathological analyses, animals were re-anaesthetized with 3.5% isoflurane, tracheotomized, artificially ventilated, and perfusion-fixed with phosphate-buffered 4% formaldehyde after 7 days of reperfusion. The brains were allowed to fix *in situ* for one hour and were then removed, dehydrated, and embedded in paraffin. The paraffin embedded brains were sectioned at 5 μm, and stained with celestine blue and acid fuchsin. Sections were deparaffinized, incubated in celestin blue solution (0,5%) for 1 minute and after washing with acetic acid incubated in acid fuchsin solution (1%) for 1 minute. After dehydration and mounting, brain damage was quantified by visual counting of intact violet-stained neurons in a blinded manner according to Nellgard and Wieloch [17]. Undamaged neurons, which had an intact nucleus and stained violet, were counted in three defined fields, each 400 μm in length, of the CA1 subsector of the dorsal hippocampus (3.8 to 4 mm caudal to bregma): the medial, hipp (1), the middle, hipp (2), and the lateral, hipp (3) CA1 (at the border to CA3). Cortical (Cx) damage was assessed in a circular area of 400 μm in diameter of the temporal cortex which is most sensitive area in the model of global ischemia. Striatal (Str) damage was assessed as neuronal score: score 0 - no damage, score 1 - damage to dorsal striatum or ventral striatum, 2- both ventral and dorsal rim, 3- entire striatum affected.

### *TUNEL* assay

The assay was performed according to the manufacturers instructions using the protocol for paraffin-embedded tissue sections (Roche Diagnostics GmbH, Germany). TUNEL positive cells were counted from the entire CA1 region of the hippocampus (approximately 3.8 to 4 mm caudal to bregma) and presented as absolute numbers.

### Behavioral tests

This test combines a sensori-motor test and a memory test of the T-maze type [18]. The rotating pole test measures coordination and integration of movements [19]. Rats traverse an elevated wooden pole (elevation 700 mm, diameter 40 mm, length 1500 mm) rotating at a speed of 10 rpm to the left or right. Prior to ischemia the animals are trained several days before ischemia until they can cross the pole onto a platform and enter the home cage. The home cage, made of transparent plexi-glas with an entrance opening at the left edge, is placed in front of the platform at the end of the pole. A cardboard wall is placed perpendicular to the cage wall, immediately to the right of the rotating pole with the entrance accessible, but not seen by the rat. This part of the test requires retention of memory. At 7 days after ischemia, each animal was tested twice, while recorded by a video camera. A score (0 to 6) was subsequently assigned to each performance. 0 means falling off the pole immediately; 1 means unable to traverse the pole; 2 means falling off the pole while crossing; 3 denotes crossing the pole while slipping and jumping with the hind limbs; 4 means crossing the pole with at least 50% slips; 5 means crossing with few slips; 6 indicates crossing with no foot slips. Score 5–6 is considered as normal performance. Correct or failed entry into the homecage were registered.

### Statistical analysis

Differences in the number of cells in the hippocampus and cortex in saline and EPO treated rats were analyzed by the Students *t*-test. Effects of EPO treatment on neuronal survival in the striatum were analyzed by the Mann–Whitney test. Individual physiological parameters between saline and EPO treated animals at the same time point were analyzed by the Students *t*-test. Physiological parameters within a treatment group were analyzed by ANOVA for repeated measurements and Dunnetts T3 posthoc test. Behavioral tests were anlyzed by the Mann–Whitney test. Statistical analyses were performed using IBM SPSS Statistics 20.0 (IBM Svenska AB, Sweden).

## Results

### Physiological parameters and serum EPO levels

**Figure 1:**
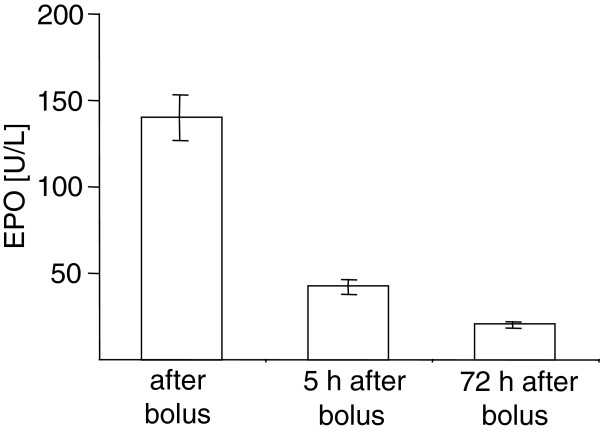
**Serum EPO concentrations after EPO treatment.** Serum EPO levels measured after bolus injection of 1600 IU and 5 and 72 hours after EPO treatment (see Methods section). Data are presented as mean ± SEM and obtained from 9 rats.

Table 1 shows physiological data obtained from rats of both treatment groups during ischemia and the subsequent three days after the insult. As indicated, a decrease of hemoglobin and hematocrit was observed in saline treated rats but remained stable in EPO treated rats after ischemia. Measurement of serum EPO levels revealed a fast increase after bolus injection of EPO into the jugular vein. Levels remained detectable until the end of the treatment period three days after ischemia (Figure 1).

**Table 1:** Physiological parameters of rats subjected to ischemia

	**Treatment**	**Before occlusion**	**After recirculation**	**After bolus injection**	**5 h after ischemia**	**72 h after ischemia**
mean arterial pressure (mmHg)	vehicle	123.4 ± 22.1	125.1 ± 21.8	123.7 ± 14.3		
	EPO	111.0 ± 9.7	116.75 ± 15.3	103.3 ± 11.8		
temperature (°C)	vehicle		37.82 ± 0.3			38.05 ± 0.5
	EPO		37.49 ± 0.9			37.62 ± 0.5
pCO_2_ (kPa)	vehicle	4.82 ± 0.96	5.55 ± 1.01	4.73 ± 0.6	5.77 ± 0.41	5.92 ± 0.48
	EPO	5.26 ± 1.45	5.75 ± 1.1	5.28 ± 1.89	5.79 ± 0.63	6.35 ± 0.66
pO_2_ (kPa)	vehicle	17.37 ± 2.57	17.05 ± 3.99	16.67 ± 3.08	16.84 ± 5.33	15.15 ± 2.05
	EPO	17.86 ± 2.64	17.69 ± 2.58	17.61 ± 3.05	17.4 ± 4.53	15.23 ± 2.77
pH	vehicle	7.43 ± 0.07	7.34 ± 0.15	7.47 ± 0.06	7.42 ± 0.04	7.44 ± 0.02
	EPO	7.43 ± 0.07	7.43 ± 0.06	7.46 ± 0.1	7.43 ± 0.03	7.43 ± 0.05
HCO_3_^-^	vehicle	24.32 ± 0.87	26.02 ± 2.72	26.27 ± 1.76	27.1 ± 1.69	29.0 ± 0.7
	EPO	25.88 ± 1.39	27.16 ± 1.38	27.68 ± 0.92	27.60 ± 1.05	29.36 ± 1.53
Hb (g/l)	vehicle	159.8 ± 6.61	148.0 ± 11.79	152.0 ± 9.12	145.57 ± 9.45	125.67 ± 4.73*
	EPO	149.57 ± 8.1	133.86 ± 9.99	147.0 ± 5.24	141.67 ± 13.49	132.46 ± 27.52
HCT (%)	vehicle	48.9 ± 2	45.4 ± 3.5	46.6 ± 2.8	44.6 ± 2.8	38.7 ± 1.5*
	EPO	45.0 ± 3.2	41.8 ± 2.6	44.2 ± 3.1	42.7 ± 4.5	42.3 ± 5.4
glucose (mmol/l)	vehicle	5.88 ± 2.7				
	EPO	7.05 ± 1.6				
	treatment	before occlusion				7 days after ischemia
body weight (g)	vehicle	332.0 ± 8.3				335.1 ± 17.5
	EPO	345.0 ± 18.3				332.9 ± 18.9

Data are presented as means ± standard deviation at the time of recirculation. Statistical differences are indicated. No indications are given if no difference was observed between the treatment groups. *p = 0.0002 vs vehicle treatment before occlusion.

### Brain damage

**Figure 2:**
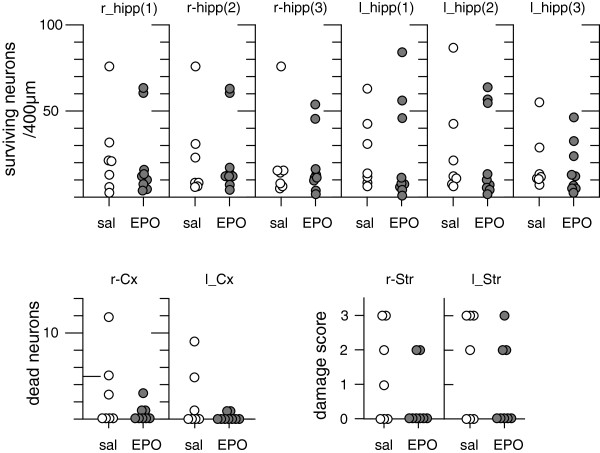
**Assessment of neuronal survival after global ischemia.** Neuronal damage in three areas (lateral (1), mid (2) and medial (3)) of the dorsal rat hippocampus (hipp) expressed as percent of undamaged cells obtained in controls, the neocortex (Cx) and in the striatum (Str) as damage scores. Each dot represents an individual animal. Animals were treated with either vehicle or EPO infusion of 3 days. Damage was assessed on day 7 after 10 minutes of global cerebral ischemia. No significant difference between vehicle or EPO treated groups was found. Statistical analysis: hippocampus and cortex – Students *t*-test; striatum - Mann–Whitney test. Abbreviations: l –left, r – right, sal – saline.

**Figure 3:**
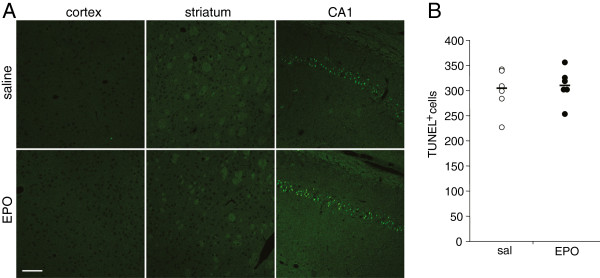
**Effect of EPO treatment on delayed cell death after global ischemia.** (**A**) Representative micrographs of coronal sections showing no TUNEL positive cells in the cortex and striatum. TUNEL positive pyramidal neurons were observed in the CA1 region of the hippocampus in both treatment groups. (**B**) Quantification of TUNEL positive cells in the entire CA1 region of the hippocampus displayed as absolute numbers with the median (black line), p = 0.69, Students *t*-test.

Brain damage was assessed in the hippocampal CA1 region, the cortex and striatum. In control animals neurons appear as large violet stained cells with a diameter of 30-50 μm and with a large owls eye-like nucleus. Damaged neurons were triangular shaped red-pink stained, with condensed dark nuclei as described previously [20]. Ten minutes of global brain ischemia induces a mean 80–90 percent damage to the dorsal aspect of the CA1 region with some animals with less damage (40-50%). Damage to cortex is less pronounced at this short time of ischemia, and hence few damaged neurons were detected. In striatum, damage is variable affecting the dorsal and ventral part as well as the entire striatum and therefore sensitive to any modulation of neuronal survival by EPO. Statistical analysis did not reveal any significant difference between saline treated (n = 7) and EPO treated (n = 9) animals on cell survival (CA 1 region) or cell death (striatum and cortex) (Figure 2). In addition, EPO treatment did not affect the number of apoptotic cells in the CA1 region of the hippocampus (Figure 3). Moreover, no TUNEL positive cells were found in the neocortex and striatum. Data show that delayed cell death is induced in the CA1 region of the hippocampus after global ischemia, without being affected by EPO treatment.

### Sensori-motor and memory performance

**Figure 4:**
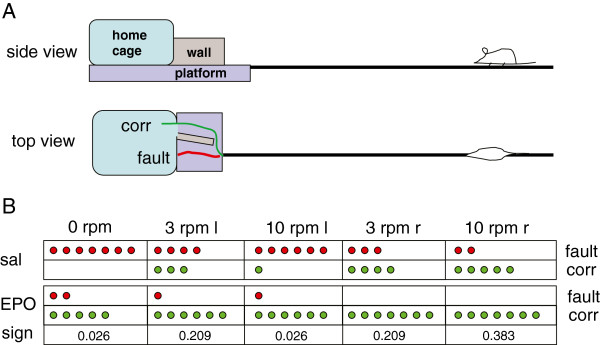
**memory retention test.** (**A**) Design of the test: animals crossed a rotating pole at 0, 3 or 10 rpm either to the right or to the left. At the end of the pole the rat were allowed to find a hidden entrance and the successfull (1) or failed (0) entries noted. (**B**) Scores of rats subjected to ischemia and treated either with saline (n = 7) or EPO (n = 7) for 3 days. Mann–Whitney test, individual significances are shown in the Figure. Abbreviations: l –left, r – right, sal – saline, sign - significance.

All experimental animals scored 5–6 on the rotating pole part of the test, which is considered normal, and also obtained in sham operated rats. In the memory challenging part of this test, saline treated animals generally failed to enter the home cage. When the pole was rotating to the left 4 out of 7 animals failed when the pole was rotating with 3 rotations per minute (rpm) and 6 out of 7 animals failed at 10 rpm, respectively, and when rotated to the right, 2 out of 6 succeeded finding the entry at 10 rpm. In contrast, all EPO treated animals, 7 out of 7, successfully and directly entered the cage when the pole rotated to the right, and 6 out of 7 when the pole rotated to the left (Figure 4). The test was performed repeatedly during test sessions. Therefore, the results indicate a spatial learning deficit in rats treated with saline after ischemia.

## Discussion

This study was conducted to investigate neuroprotective effects of EPO treatment in rats subjected to global ischemia and sought at identifying if the treatment is associated with an improvement of sensorimotor and memory function. We show that EPO has no influence on neuronal survival and delayed neuronal cell death in the CA1 region in the hippocampus, cortex and striatum of rats subjected to ischemia but treatment significantly preserved memory function.

### Pharmacokinetics of EPO delivery and long-term effects on blood parameters

Administration of EPO by continuous infusion ensured serum levels similar to those found in animals subjected to intermittent hypoxia [21]. Interestingly, we also observed a reduction of hemoglobin and hematocrit in saline treated animals but not in EPO treated rats within the first 72 hours after ischemia. Postischemic EPO treatment has led to increased erythropoiesis compensating the loss of red blood cells related to the ischemia model. Thus, it might be anticipated that beneficial effects of EPO on memory function might be related to the prevention of postischemic anemia. Deleterious effects of anemia on stroke outcome have been described in patients [22], hence, previous studies also have shown that EPO mediated effects on memory function are independent from its effects on the erythropoietic system [23].

### Effects of EPO on neuronal damage after global ischemia

Our results confirm those of Popp and colleagues [15] showing lack of neuroprotective efficacy of EPO in a rat model of cardiac arrest imposing a 8 minutes ischemic insult to the brain. In addition, we did not find any changes in cell death in the striatum, a region of rapidly progressing damage [24]. These results contrast those obtained when EPO was administered intraventricularly (icv) prior to or 20 minutes after global ischemia the rat [14] or immediately upon reperfusion in the gerbil [25]. The neuroprotective effect seen after icv injections of EPO might suggest a mechanism active early (hours) of reperfusion. The present model is amenable to neuroprotection. The most robust neuroprotection is provided by mild hypothermia (body temperature 33°C) in the hippocampus and cortex when induced as late as 12 hours after reperfusion, presumably acting on multiple detrimental mechanisms. Pharmacological neuroprotection, which is not due to concomitantly induced body hypothermia [26], has a more narrow therapeutic time window. For example, cyclosporin A and the AMPA-receptor blocker NBQX are neuroprotective when administered within the first 30 min to 2 hours of reperfusion but not later [27].

Since EPO has been proposed to act both on neurons and astrocytes, by preventing apoptosis and stimulating trophic factors [28] treatment would be expected to be hypothermia-like, i.e. act on several mechanisms thereby providing protection. To what extent EPO treatment contributes to the release of pro-inflammatory cytokines and other inflammatory actions of microglia/macrophages after ischemia has to be addressed in future studies [29,30]. Evidently, the EPO targeted detrimental mechanisms do not appear to contribute to selective and delayed neuronal death after global ischemia. In contrast, EPO treatment effectively prevents pan-necrosis of the penumbra tissue after focal ischemia.

### Effects of EPO on sensori-motor and memory functions

Global ischemia is known to induce relative subtle behavioral deficits. Here we present a novel and simple test for combined non-associative factors (sensori-motor deficit), with memory retention. Loss of memory function in the 2-VO model of global ischemia has earlier been seen in the radial maze-tests and Morrize water maze test, probing spatial learning and retention of working memory [31]. Our data showing retained “cognitive” function in animals injured by global ischemia is striking. This is in line with the findings that EPO enhances LTP and memory functions in naive rats [13] and improves memory function in a model of Alzheimers disease [32] and long-term spatial memory deficits and brain injury following neonatal hypoxia-ischemia in rats [33]. In that context high expression of EpoR in pyramidal neurons in the cortex and hippocampus has been associated with higher cognitive functions [34]. Erythropoietin treatment also increased the hippocampal response during picture encoding and retrieval in human [23] and increased expression of the EpoR has been found in postmortem analyses of patients with Alzheimers disease [35].

In focal ischemia treatment with EPO 24 h after reperfusion enhances recovery of neurological function without affecting infarct size [36]. This suggests that EPO enhances recovery of transmission that is lost during brain ischemia. The recovery enhancing action of EPO could be an effect on synaptic function of pre-existing synapses on surviving neurons, or by formation of new synapses on surviving neurons. Since the animals learned the task prior to ischemia, our study design probes retention of memory traces on existing synapses, rather than formation of new synapses, which would require a period of re-learning. Since damage is almost 70-90% in the dorsal hippocampus, we are inclined to support the notion that EPO prevents the loss of memory in the ventral parts of hippocampus, and enhances LTP in this region.

## Conclusions

We conclude that intravenous EPO administration after global ischemia does not protect against ischemic brain damage, but protects against loss of synaptic function important for working and spatial memory. We believe our data have important clinical implications, and propose that EPO treatment may be beneficial for the retention of cognitive functions of the cardiac arrest patient.

## Competing interests

The authors declare that they have no competing or conflicting interests.

## Authors’ contributions

JU and CS performed the animal experiments. JKL has analyzed the blood samples. TW together with BR wrote the manuscript. KR performed the TUNEL assay and revised the manuscript. BR conceived the study. All authors read and approved the final manuscript.
